# A Second-Generation Voltage-Conveyor-Based Interface for Ultrasonic PVDF Sensors

**DOI:** 10.3390/mi12020099

**Published:** 2021-01-20

**Authors:** Salvatore A. Pullano, Antonino S. Fiorillo, Gianluca Barile, Vincenzo Stornelli, Giuseppe Ferri

**Affiliations:** 1 Department of Health Sciences, University “Magna Græcia” of Catanzaro, 88100 Catanzaro, Italy; pullano@unicz.it (S.A.P.); nino@unicz.it (A.S.F.); 2 Department of Industrial and Information Engineering and Economics (DIIEE), Faculty of Engineering, University of L’Aquila, 67100 L’Aquila, Italy; vincenzo.stornelli@univaq.it (V.S.); giuseppe.ferri@univaq.it (G.F.)

**Keywords:** sensor interface, ultrasonic transducer, PVDF, voltage conveyor

## Abstract

Exploiting the transmission and reception of low frequency ultrasounds in air is often associated with the innate echolocating abilities of some mammals, later emulated with sophisticated electronic systems, to obtain information about unstructured environments. Here, we present a novel approach for the reception of ultrasounds in air, which exploits a piezopolymer broadband sensor and an electronic interface based on a second-generation voltage conveyor (VCII). Taking advantage of its capability to manipulate both voltage and current signals, in this paper, we propose an extremely simple interface that presents a sensitivity level of about −100 dB, which is in line with commercially available references. The presented results are obtained without any filtration stage. The second-generation voltage conveyor active device is implemented through a commercially available AD844, with a supply voltage of ±15 V.

## 1. Introduction

In the most general accepted understanding of the term, a sensor is a device that provides a quantified electrical response by sensing the changes in a chemical, physical, biological, etc., phenomenon. In nature, these changes are often slow and continuous, and commonly defined as analog [1,2]. To design the most appropriate interface (i.e., the one that increases the system sensitivity), the design of a suitable electrical model of the sensor and the knowledge of the electrical parameter that changes are highly desirable. A properly conceived electronic interface avoids the loading effects related to the transducer-interface impedance and is adapted to the entity of input variations (small or large) that the sensor detects. Therefore, measurement and information are crucial to the designer to make preliminary choices, such as powering the sensor in alternating current (AC) or direct current (DC), or designing the interface using discrete components or integrated custom circuits.

As a first approach, a discrete circuit topology could be designed. If the solution is satisfactory, it is possible to design the corresponding integrated version. Complementary metal oxide semiconductor (CMOS) technology allows for the integration of electronic interfaces operating at a lower supply voltage and power consumption and reduces side-effects of noise, offset, and its environmental dependence (e.g., temperature). If the sensor response is sufficiently slower than the dynamic of the interface, the slew rate or bandwidth may not be real constraints [3,4,5,6]. The sensor system development (sensor and interface) can share the same chip and fabrication technology, as in the microsystem approach, or can be designed and fabricated separately, as in micromodules.

Among the most investigated sensors, piezoelectric sensors have been widely investigated, especially for their generation and reception of ultrasounds in different fields such as echolocation and communication systems, medical treatment, and nondestructive evaluations, etc. [7,8,9]. They exploit the surrounding air as the coupling medium between the transmitter and the receiver. According to the analysis of the main features of the ultrasonic signal (e.g., time-of-flight, attenuation, or spectrum), specific information can be retrieved from the environment [10]. To date, a plethora of materials and specific geometries have been proposed to address the problem of matching the acoustic impedance in the air. Materials such as lead–zirconate–titanate (PZT)-based devices, electromechanical film (EMFi), piezopolymers, and different composites were investigated to this end [11]. The piezopolymer polyvinylidene-fluoride (PVDF) was used to manufacture curved transducers for short-range robotic applications, due to its lightness, flexibility, low quality factor, and its good acoustic impedance matching with air [12]. A spiral-shaped PVDF transducer was recently investigated, inspired by the cochlea of mammals, which can be considered one of the most sophisticated and fascinating components of a natural echo localization system. It was developed to guarantee uniform sensitivity on the vertical (XY) and horizontal (XZ) planes, omnidirectionality, and a wide frequency band between 30 and 95 kHz [13].

The charge or voltage shown by the piezoelectric element is mostly acquired through two different read-out circuits: a high impedance preamplifier (voltage mode) or a low input impedance preamplifier (charge mode) [14]. Typically, preamplifiers are designed using operational amplifiers (Op-Amps), as an active block. Even though widely employed, its use leads to an electronic interface with high complexity, low sensitivity at low supply voltages, and low bandwidths, which are, in turn, constrained by a constant gain bandwidth product. In this paper, we present a novel alternative to standard Op-Amp transconductance amplifier, exploiting a current mode approach based on a second-generation voltage conveyor (VCII) [15,16,17]. Unlike Op-Amp, VCII allows the achievement of a constant bandwidth at different gain levels while maintaining full compatibility with a voltage mode signal processing chain and offering a low impedance voltage output port. Its main feature is a current input terminal with low impedance, virtually grounded (which allows directly interfacing the spiral-shaped PVDF sensor), which converts the output current into a proportional voltage. Here, we present a spiral-shaped PVDF sensor designed and fabricated to receive ultrasounds in air in a wide frequency range with omnidirectional sensitivity. Then, we introduce the proposed electronic interface based on VCII, with an emphasis on the design procedure and with the aid of critical simulation results. Finally, experimental results of the fabricated sensor system are presented and compared with a standard electronic interface.

## 2. Materials and Methods

### 2.1. PVDF Ultrasonic Sensor

First attempts to use PVDF film to transmit ultrasounds in air date back to 1975 by using a cylindrical geometry and working in 3–1 mode [18,19,20]. The arrangement allowed the conversion of the PVDF displacement into a radial vibration, characterized by a resonant frequency (*f_r_*), which is related to the bending radius (*r*) according to the following relationship, where *ρ* is the mean density of the metallized film, and *Y* is the Young’s modulus [9]:

(1) $$f_{r}=\frac{1}{2\pi r}\sqrt{\frac{Y}{\rho }}$$

Based on the theory of curved PVDF, different geometries have been proposed for improving the performances in terms of resonance frequency, bandwidth, and in air coupling, as evidenced in Table 1.

**Figure 1:**
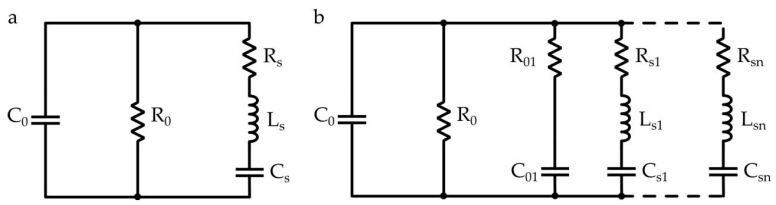
(**a**) Hemi-cylindrical PVDF equivalent model based on the BVD theory. (**b**) Spiral-shaped PVDF modified BVD model considering multiple resonances.

PVDF-based ultrasonic (US) transducers are characterized by a low-quality factor (Table 1), as well as low acoustic impedance (more than one order of magnitude compared to standard non-polymeric piezoelectric materials) [11]. Moreover, most PVDF sensors suffer from a narrow band (not more than 10 kHz), which is not fully suitable for accomplishing all the tasks required by some specific applications (e.g., mobile robots). From the electric point of view, hemi-cylindrical PVDF film was modeled according to the Butterworth–Van Dyke (BVD) model, composed of a low frequency branch with a capacitor *C*_0_, shunted by a resistor *R*_0_; this considers the dielectric losses and a series *R_S_L_S_C_S_* resonant branch (Figure 1a). More recently, spiral-shaped geometries, commonly found in nature, were investigated, evidencing the coexistence of multiple vibrations modalities (radial and flexural resonances), which allow describing the transducer as a summation of hemi-cylindrical resonators with contiguous resonance frequencies (Figure 1b) [13,20,23]. The latter design is expected to comprise multiple hemi-cylindrical resonators distributed along the whole length of the PVDF sheet. Therefore, the whole geometry can be approximated by multiple series of RLC resonators leading to multiple contiguous bands that collectively support a US transducer with a broader frequency range, increasing the performance achievable through a single device. The static branches *C*_0_ and *R*_0_ vary considerably with frequency, even more considering a broad frequency range. To consider this frequency dependence, a modified BVD model was proposed by introducing a series of a resistors *R*_01_ and a capacitor *C*_01_ (see Figure 1b) [24].

**Figure 2:**
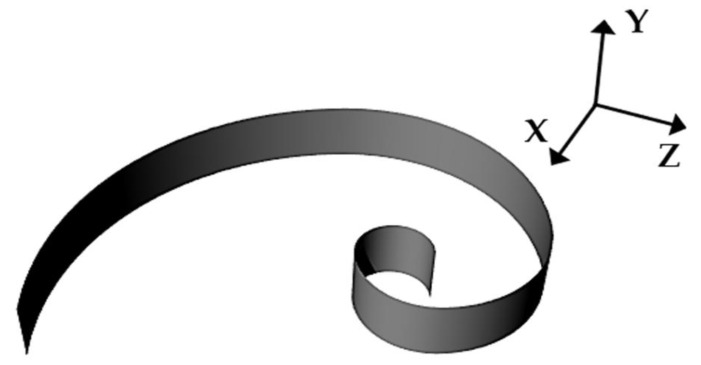
Spatial arrangement of the PVDF sensor.

In this case, the model was simulated considering frequency-invariant components in the range 1–150 kHz. The RLC lumped parameters of the PVDF electrical model in Figure 1a can be evaluated through the admittance Y (modulus and phase) according to a procedure suggested by Brown et al. [25]. The proposed sensor was designed according to a logarithmic spiral with the aim of receiving ultrasonic signals in a frequency range from 20 to 80 kHz (Figure 2).

We used thin 15 × 5 mm PVDF sheet (TE Connectivity’s (TE) Measurement Specialties, Hampton, NY, USA) with a thickness of 28 µm. The film was silver metallized on both surfaces, working in 3–1 mode, with a transverse piezoelectric constant of d_31_ = 21 pC·N^−1^ and a Young’s modulus of about 4 GPa. Subsequently, the PVDF film was folded according to a logarithmic spiral geometry. Logarithmic dependence of radial distance *r* respect on the angular position *φ*, according to the relation ln(*r*) = ln(*a*) + *φ b*, with *a* and *b* being arbitrary constants that allow modifying the shape of the curve. The obtained geometry was characterized by arc lengths in the range between 6 and 25 mm, corresponding approximately to the frequency range reported above.

The folded thin film was clamped at the extremities maintaining the film free to vibrate. The proposed PVDF device is bimodal so it can work both as a transducer (for the generation of ultrasounds in air) and as a sensor (for the reception of incoming ultrasounds in air).

### 2.2. Electronic Interface

**Figure 3:**
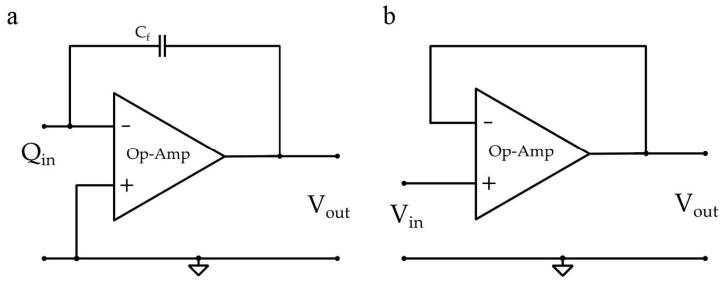
(**a**) charge mode and (**b**) voltage mode Op-Amp based standard interfaces.

The charge or voltage generated by piezoelectric element is mostly acquired through two different read-out circuits: in the case of charge mode (Figure 3a), the low input impedance of the charge amplifier is preferred, whereas the high impedance of voltage preamplifier, connected in a unity gain configuration, is used in the case of voltage mode (Figure 3b) [14].

For these kind of sensors, although using Op-Amps for signal processing has proved to be a flexible solution [26,27,28,29], electronic interfaces based on this active block inherit several well-known limitations, such as a finite gain-bandwidth product (GBW) and a relatively complex transistor-level topology (in the case of integrated solutions). The latter implies a high design effort to fulfill key parameters for a sensor interface, such as low noise, high common mode rejection ratio (CMRR), and adequate terminal impedances. Moreover, when considering low-pitch integrated technologies that require very low supply voltages, it becomes challenging to achieve sufficient sensitivity (and hence resolution) for any given application [30,31,32].

**Figure 4:**
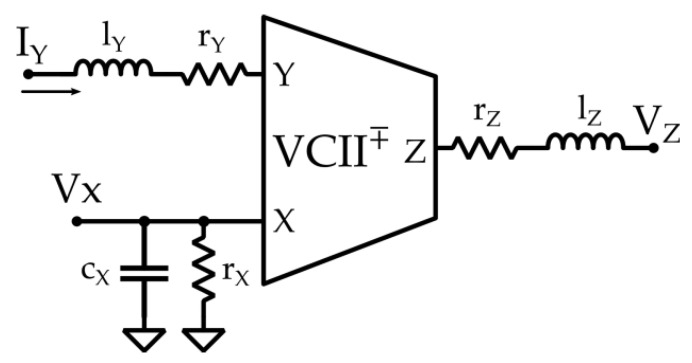
VCII high-level model including node parasitic impedances.

In this regard, a current mode signal processing can overcome the aforementioned limitations, since current mode active devices (such as second-generation current conveyors (CCIIs)) are typically simpler and more energy efficient. Moreover, these devices can operate in open loop configuration, making it unnecessary to perform a frequency compensation. This allows achieving a variable GBW or, in other words, the possibility of obtaining high gain levels without affecting the bandwidth of the electronic interface. One of the main downsides of using CCIIs is their lack of a low impedance voltage output terminal, making it cumbersome to use in a voltage mode processing chain [33,34,35,36,37,38]. Recently, a novel mixed-mode analog active block, named Second-generation voltage conveyor (VCII), has been proposed to overcome that limitation [15,16,17] and used in many applications [39,40,41,42,43]. As its name suggests, it can be considered the dual version of a CCII; it is a three terminal building block (Figure 4) whose terminal cross-relationships are fully described by the following matrix, which includes non-ideal parasitic impedance parameters on its terminals:

(2) $$\left[\begin{array}{c} i_{x}\\ v_{y}\\ v_{z} \end{array}\right]=\left[\begin{array}{ccc} \frac{1}{r_{x}//\left(1/sCx\right)} & \pm \beta & 0\\ 0 & r_{y}+sL_{y} & 0\\ \alpha & 0 & r_{z}+sL_{z} \end{array}\right]\left[\begin{array}{c} v_{x}\\ i_{y}\\ i_{z} \end{array}\right]$$

From Equation (2), it is possible to make the following considerations: The Y terminal is a current input; therefore, its impedance should ideally be equal to zero. As a consequence, this terminal represents a virtual ground, that is *V_y_* = 0. Unlike Op-Amps, the virtual ground feature is obtained without the need for a negative feedback loop, and this is beneficial in applications where the sensor has a high output impedance. The current fed into the Y terminal is conveyed to the X terminal, which, in turn, is a high (ideally infinite) impedance voltage input. The β parameter, in this case, represents the mirroring coefficient and should be as close to unity as possible. Notably, it can be both positive and negative, determining whether the current in the X terminal flows in the same direction (positive, VCII+) or in the opposite direction (negative, VCII−) with respect to that related to the Y terminal. The voltage produced at the X terminal is buffered to the Z terminal, which is a low impedance (ideally zero) voltage output. This feature ensures that, unlike CCIIs, this building block is natively compatible with a voltage mode signal processing environment. The α parameter represents the voltage buffering coefficient, and, again, should be designed to be equal to unity. Considering Figure 4 and Equation (2), *r_y_*, *L_y_*, *r_x_*, *C_x_*, *r_z_*, and *L_z_* represent the main parasitic elements the related terminals. In the ideal scenario, they are equal to zero, except for *r_x_*, which is equal to infinity.

**Figure 5:**
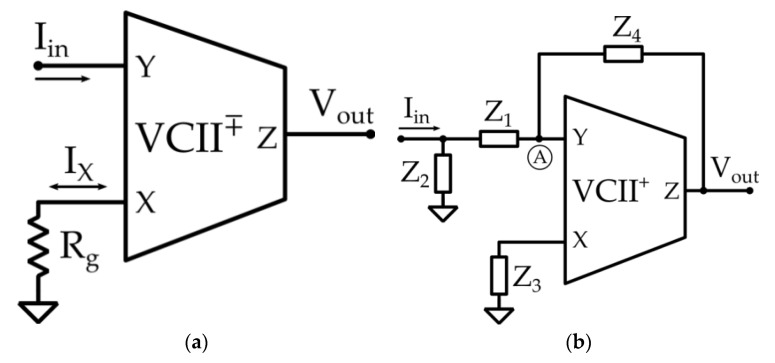
(**a**) VCII-based transimpedance amplifier, and (**b**) VCII-based reconfigurable second-order low-pass and bandpass filter.

The proposed interface is shown in Figure 5. It is composed of two stages (channels), which can be used to alternatively interface a single sensor, or to simultaneously interface a couple of sensors (Section 3). The first channel is a VCII-based transimpedance amplifier (Figure 5a); it is a simple circuit and can be used when there is no need to perform a filtering action on the input signal. Conversely, the second channel is a reconfigurable second-order low-pass or bandpass filter (Figure 5b). It is a more complicated solution but offers the flexibility to perform a transimpedance conversion while operating a filtering action to the input signal.

Considering the configuration proposed in Figure 5a, making the reasonable assumption that *r_x_ >> R_g_*, and from Equation (2), we can express the voltage at the *X* terminal, given *I_in_* at the *Y* terminal as:

(3) $$V_{x}=I_{x}R_{g}=\pm \beta I_{in}\left(R_{g}//r_{x}\right)≅\pm \beta I_{in}R_{g}$$

Considering the voltage buffering action between *X* and *Z* terminals, the output voltage at *Z* is defined by the following equation:

(4) $$V_{out}=V_{z}=\alpha V_{x}=\pm \beta \alpha R_{g}I_{in}$$

If *α* and *β* parameters are sufficiently close to unity, it is possible to neglect them, obtaining a direct current-to-voltage conversion whose transimpedance gain only depends on the gain resistor *R_g_*.

Considering the filtering circuit depicted in Figure 5b, depending on the nature of the impedances *Z*_1_–*Z*_4_, it can perform either a second-order low-pass filtering operation (*R*_1_, *C*_2_, *R*_3_, *C*_4_) or a second-order bandpass action (*C*_1_, *R*_2_, *C*_3_, *R*_3_). As previously explained, the *Y* port can be virtually considered at ground, *V_Y_* ≈ 0, so it is possible to write:

(5) $$I_{Z1}=\frac{Z_{2}}{Z_{1}+Z_{2}}I_{in}$$

(6) $$V_{out}=-Z_{4}I_{Z4}$$

The current conveying action of a VCII from the *Y* terminal to the *X* terminal allows calculating the voltage at the *X* port as:

(7) $$V_{x}=-Z_{3}\beta I_{Z3}=-Z_{3}\beta I_{Y}$$

Since *V_z_ = αV_x_*, it is possible to write:

(8) $$V_{x}=\frac{1}{\alpha }V_{out}=-\frac{1}{\alpha }Z_{4}I_{Z4}$$

Combining Equations (7) and (8), we have:

(9) $$I_{Y}=\frac{Z_{4}}{\alpha \beta Z_{3}}I_{Z4}$$

By then applying the Kirchhoff current law (KCL) at A node, we can write:

(10) $$I_{Z1}=I_{Y}+I_{Z4}$$

Combining Equations (5), (9), and (10), we obtain:

(11) $$I_{in}\frac{Z_{2}}{Z_{1}+Z_{2}}=\frac{Z_{4}+\alpha \beta Z_{3}}{\alpha \beta Z_{3}}I_{Z4}$$

Considering *α = β = 1*, and combining Equations (8) and (11), it is possible to achieve a generic transimpedance transfer function for the configuration under examination:

(12) $$\frac{V_{out}}{I_{in}}=\frac{Z_{2}Z_{3}Z_{4}}{\left(Z_{1}+Z_{2}\right)\left(Z_{3}+Z_{4}\right)}$$

As previously introduced, by choosing *Z*_1_ *= 1/sC*_1_, *Z*_2_ *= R*_2_, *Z*_3_ *= 1/sC*_3_, and *Z*_4_ *= R*_4_, Equation (12) becomes the transimpedance transfer function of a second-order bandpass filter:

(13) $$\frac{V_{out}}{I_{in}}=\frac{sC_{1}R_{2}R_{4}}{1+s\left(C_{1}R_{2}+C_{3}R_{4}\right)+s^{2}C_{1}C_{3}R_{2}R_{4}}$$

By choosing *Z*_1_ *= R*_1_, *Z*_2_ *= 1/sC*_2_, *Z*_3_ *= R*_3_, *Z*_4_ *= 1/sC*_4_, Equation (12) becomes the transimpedance transfer function of a second-order lowpass filter:

(14) $$\frac{V_{out}}{I_{in}}=\frac{R_{3}}{1+s\left(C_{2}R_{1}+C_{4}R_{3}\right)+s^{2}C_{2}C_{4}R_{1}R_{3}}$$

Regardless of the filter type, the quality factor *Q* and the natural frequency *ω*_0_ are calculated as:

(15) $$Q=\frac{\sqrt{C_{1}C_{3}R_{2}R_{4}}}{C_{1}R_{2}+C_{3}R_{4}}$$

(16) $$\omega_{0}=\sqrt{\frac{1}{C_{1}C_{3}R_{2}R_{4}}}$$

### 2.3. Characterization Setup

**Figure 6:**
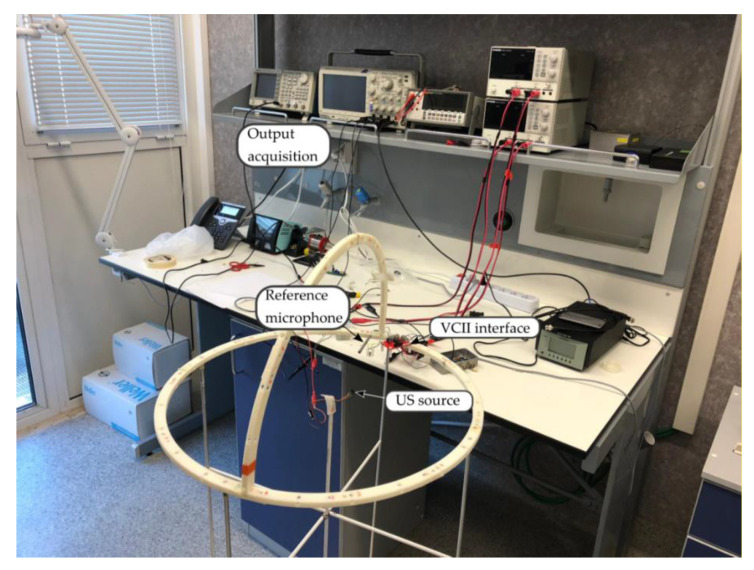
Measurement setup.

In reception, the sensitivity of a PVDF sensor can be defined at a specific distance, by considering a reference sensitivity. To this end, a calibrated microphone (Brüel & Kjær, 4939) and a charge amplifier (Brüel & Kjær, Nexus 2690) were used as a reference for the characterization of the VCII-based electronic interface. The spiral-shaped sensor and the calibrated microphone were positioned side-by-side and stimulated by the same US signal generated by a transducer placed 0.2 m from them. The PVDF transmitter was stimulated with a 150 Vpp sinusoidal burst of 10 cycles at variable frequency in the range 20–80 kHz. The US signal received by the VCII interface was acquired and recorded by a digital oscilloscope (Tektronix, Beaverton, OR, USoregon, DPO3054, Figure 6).

Sensitivity of the spiral-shaped sensor indicates the sound receiving level expressed by:

(17) $$S=20log\left(\frac{V}{V_{0}}\right)$$

where *V* is the voltage generated by the sensor, which is irradiated with a constant pressure level of 1 Pa, and *V*_0_ is a reference value set to 10 V/Pa (0 dB) [44].

## 3. Results and Discussion

**Figure 7:**
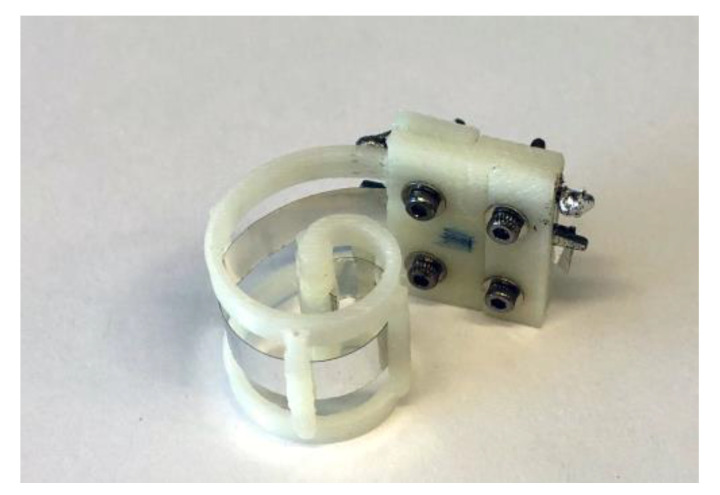
Fabricated spiral-shaped PVDF sensor clamped onto an ABS support.

The fabrication process of the PVDF sensor included the folding of thin piezoelectric film onto a 3D printed acrylonitrile butadiene styrene (ABS) support (Stratasys uPrint SE), used to impose the desired geometry, clamping the film only at the extremities, and leaving the film to vibrate freely (Figure 7). Electrical contacts were performed by micro-screws onto the two Ag metallized faces. The sensor was designed to work in 3–1 mode and thus poled in the Z-direction and folded around the *Y*-axis, according to Figure 2. The overall dimension of the sensor was about 18 mm in length and 15 mm in height. The impedance (magnitude and phase) of the sensor presented in Figure 6 was evaluated using an impedance analyzer (Keysight, Santa Rosa, CA, USA, 4980AL) in the range of 20–80 kHz, as reported in Figure 7.

**Figure 8:**
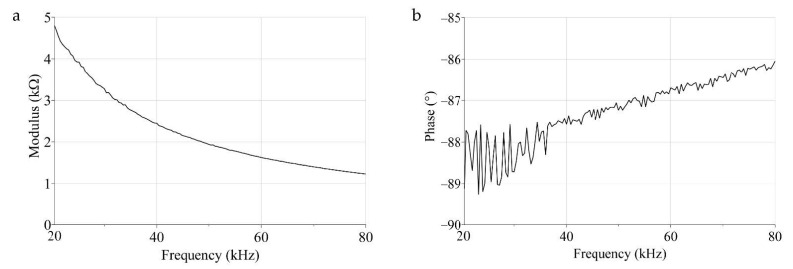
Electrical impedance magnitude (**a**) and phase (**b**) of the proposed PVDF sensor.

The magnitude (Figure 8a) evidenced a trend characteristic of a capacitive device, in accordance with the BVD model. The effect of the multiple resonance characteristics of the proposed geometry is more clearly visible on the phase, which is characterized by multiple local maxima (Figure 8b).

Impedance analysis confirmed that the spiral-shaped sensor can be seen as a continuous summation of contiguous hemi-cylindrical elements, each one characterized by a specific resonance frequency in accordance with the theory of clamped curved PVDF sensors [13]. Even though we presented a sensor shaped according to a logarithmic geometry in the proposed design, the literature reports the possibility of using other folding modes, such as Fibonacci or Archimedean [23].

**Figure 9:**
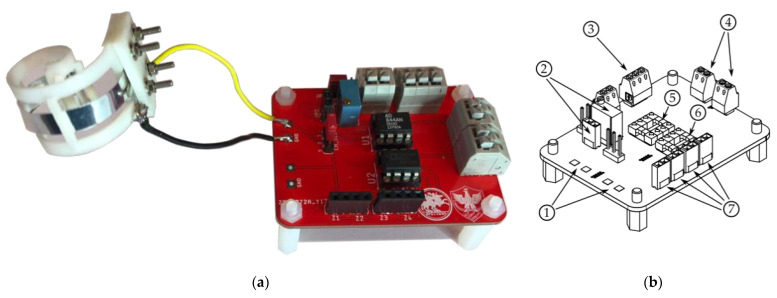

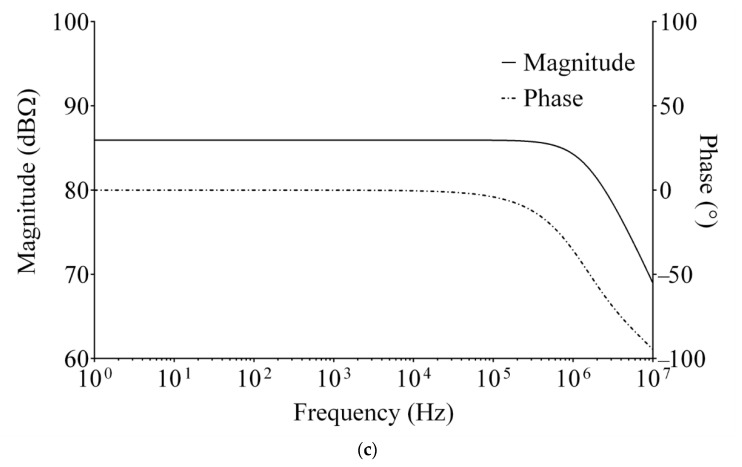
The electronic interface: (**a**) the fabricated prototype and (**b**) highlights of its components: (1) PVDF sensor inputs, (2) TIA gain regulation section, (3) supply voltage input, (4) interface outputs, (5) channel 1 VCII, (6) channel 2 VCII, and (7) filter configuration pins. (**c**) Simulated frequency behavior of the VCII-based transimpedance amplifier.

The fabricated electronic interface is shown in Figure 9a. As visible from Figure 9b, it is possible to directly interface up to two sensors and process their signal through the TI amplifier and the reconfigurable filter. The board allows directly configuring the transimpedance gain of the TIA (transimpedance amplifier) via a screw trimmer and provides the ability to freely set the filter nature. Figure 9c shows the simulated frequency behavior of the interface when using its channel 1, where the transimpedance gain was set to 20 kΩ. As visible, the magnitude remains flat across the entire band of interest.

**Figure 10:**
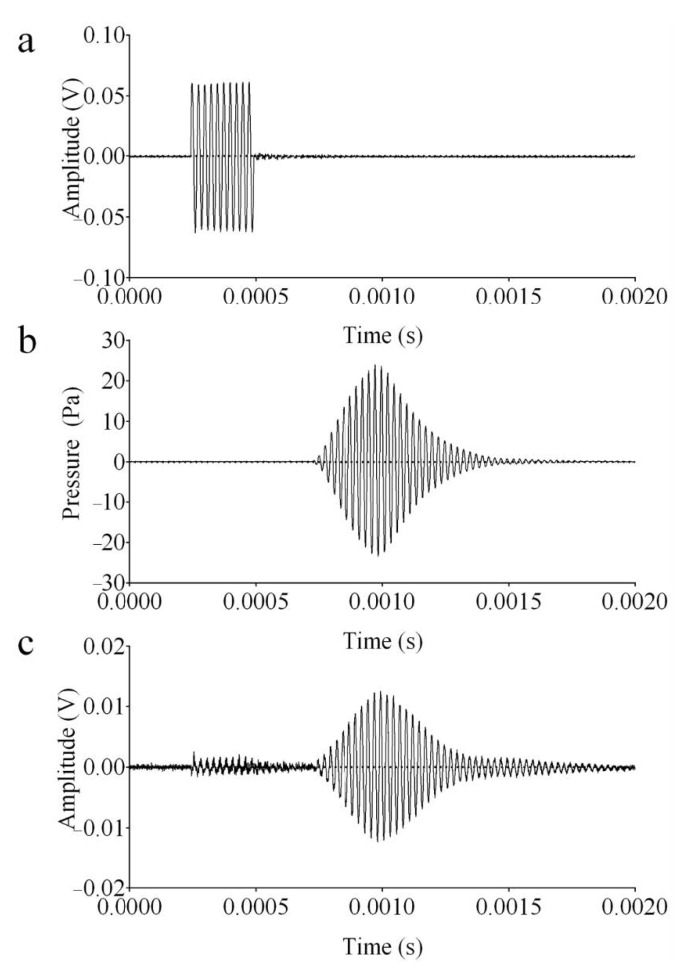
Pulse-echo signal acquisition where (**a**) is the excitation burst of the transducer, (**b**) highlights the pressure received by the spiral shaped-sensor, and (**c**) is the signal received at the output of VCII.

**Figure 11:**
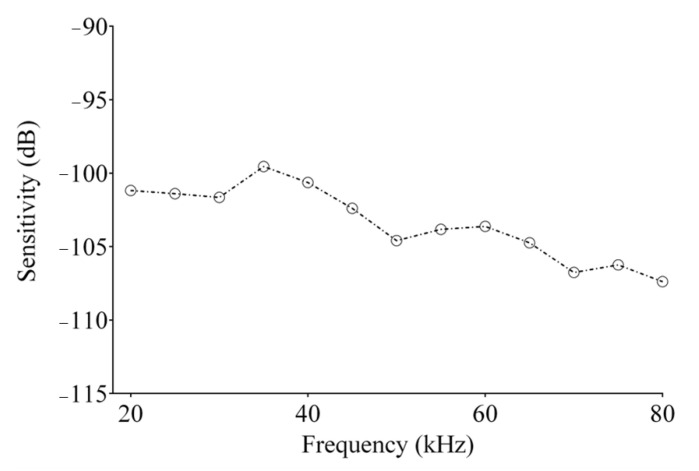
Receiver sensitivity evaluated through the VCII-based interface in the range of 20–80 kHz (10 V/Pa as 0 dB scale) on the horizontal plane with the transmitter and the received placed face-to-face.

Figure 10a shows a representative burst generated by an arbitrary waveform generator (Tektronix, AFG3102) centered at 40 kHz, which is firstly amplified (60 dB) before driving the US transmitter. Figure 10b reports the pressure received by the calibrated microphone set with a sensitivity of 10 mV/Pa, showing an acoustic pressure in the range −25 to 25 Pa. Finally, in Figure 10c, the signal obtained at the output of the VCII-based electronic interface is reported. The contribution of the electronic interface was considered as a constant gain in the band (86 dBΩ). A US signal was continuously transmitted by the broadband source and monitored by the calibrated microphone located at 0.3 m, close to the spiral shaped sensor (1 Pa root mean square (rms)). The voltage (*V_rms_*) generated by the PVDF receiver was then acquired and the sensitivity evaluated according to Equation (17). For instance, at 40 kHz, the received *V_rms_* at VCII output divided by its gain (86 dBΩ), is 96.7 μV, which corresponds, according to (17), to a sensitivity of −100.632. The sensitivity was evaluated on the horizontal plane by monitoring the received pressure by a calibrated microphone onto the spiral-shaped sensor in the range of 20–80 kHz (Figure 11). The latter shows an average sensitivity evaluated on eight independent signals acquired with a high repeatability (standard deviation < 0.2%). It evidences a variation inside the considered frequency band (20–80 kHz) in the range (−107, −101) dB which is comparable with most commercial and custom PVDF sensors used for the reception of US in air [45].

Commercial cylindrical PVDF sensors are produced only at 40 and 80 kHz, and are characterized by beam directivity on the vertical and horizontal plane of ±150° and ±40°, respectively, for the lower frequency version, and 360° and ±25° for the higher frequency one, respectively. The maximum sensitivity reported is −76 dB for the 40 kHz sensor and −90 dB for the 80 kHz sensor evaluated at the specific resonance frequency. In comparison, the proposed spiral shaped sensor can be used in a wider frequency range (20–80 kHz) with a beam directivity of 360° on both the vertical and horizontal planes, exceeding those of commercial sensors, especially on the horizontal plane. The reported sensitivity, instead, is in the range between −107 and −101 dB, comparable with the sensors fabricated with the same ferroelectric polymer technology. The proposed architecture provides relevant improvements compared to commercially available devices from the electronic interface perspective as well. Typical commercial solutions are based on the voltage mode approach, exploited via multi-staged architectures. Due to the current mode signal processing, the proposed solution achieves a larger bandwidth, ensuring negligible distortion at high frequencies. Moreover, it is able to achieve comparable SNR (Single to Noise Ratio) levels using one single active device, thereby greatly reducing power consumption and chip area occupation. Table 2 summarizes the comparison discussed so far.

## 4. Conclusions

We presented a novel electronic interface for a PVDF sensor that employs the current mode approach characteristic of the second-generation voltage conveyor. Measurements performed on the fabricated prototype evidenced the possibility of acquiring low-pressure and low-frequency acoustic signal, adopting extremely simple architectures based on the use of VCIIs. The proposed PVDF sensor is advantageously characterized by a wide frequency ranging from 20 to 80 kHz and a beam directivity of 360° on both the vertical and horizontal planes. The evaluated sensitivity is in the range of −107 to −101 dB, which is comparable with that of commercially fabricated PVDF sensors. Due to the use of novel approach based on the use of a VCII electronic interface, a larger bandwidth (>10^3^ at dBΩ) with a power consumption of 6 mA is achieved by a single-stage pre-amplifier. The results achieved in this study are relevant because they acknowledge the validity of the approach through extremely-low-voltage and low-power solutions that retain very good sensitivity.

## Figures and Tables

**Table 1:** Characteristics of representative PVDF ultrasonic sensors for in air applications.

Geometry	*f_r_* (kHz)	Bandwidth (kHz)	Quality Factor	Ref.
Hemi-cylindric	63.5	6.3	≅10	[13]
Cylindric	40	Tx (Transmission): 8	Tx: 5	
		Rx (Reception): 10	Rx: 10	[21]
Cylindric	80	Tx: 14	Tx: ≅10	
		Rx: 11	Rx: ≅7	[21]
Truncated Conical	33	11	3	[22]

**Table 2:** Performance of the proposed VCII-based electronic interface compared to other commercially available.

Sensor	Active Device	Number of Processing Stages	Filtering Stage	Gain	Bandwidth (kHz)	Power Consumption (mA)
Cylindric 40 kHz	MOS Stage	3	Bandpass	31 dB	≅100	30
Cylindric 80 kHz	Op-Amp Stage	3	Bandpass	61 dB	67	12 *
This Work	VCII	1	None	86 dBΩ	>103	6

* Estimated.
